# Exploring the Larvicidal and Repellent Potential of Taurus Cedar (*Cedrus libani*) Tar against the Brown Dog Tick (*Rhipicephalus sanguineus* sensu lato)

**DOI:** 10.3390/molecules28237689

**Published:** 2023-11-21

**Authors:** Samed Koc, Zeynep Nur Gultekin, Sevval Kahraman, Aysegul Cengiz, Burak Polat, Cansu Caliskan, Tolga Yildirim, Ozge Tufan-Cetin, Huseyin Cetin

**Affiliations:** 1 Department of Biology, Faculty of Science, Akdeniz University, Antalya 07070, Türkiye; zeynepnurgultekin@gmail.com (Z.N.G.);; 2 Laboratory Animals Application and Research Centre, Akdeniz University, Antalya 07070, Türkiye; 3 Department of Environmental Protection Technology, Vocational School of Technical Sciences, Akdeniz University, Antalya 07070, Türkiye; ozgetufan@akdeniz.edu.tr

**Keywords:** acaricidal, *Cedrus libani*, larvicidal, repellent, tar, ticks

## Abstract

This study investigated the potential acaricidal and repellent effects of tar obtained from the Lebanon cedar (*Cedrus libani* A. Rich.) against the brown dog tick species *Rhipicephalus sanguineus* sensu lato Latreille (Acari: Ixodidae). The goal was to find an alternative, safe, and effective way to eliminate ticks. Tar is traditionally extracted from cedar trees in the Antalya region of Türkiye. The composition of the tar is primarily characterized by a diverse mixture of terpenes, with β-himachalene (29.16%), α-atlantone (28.7%), ar-turmerone (8.82%), longifolene-(V4) (6.66%), α-himachalene (5.28%), and β-turmerone (5.12%) emerging as the predominant constituents. The toxic effects of tar on tick larvae were studied through larval immersion tests (LIT), and its repellent activity was evaluated using a new larval repellent activity test (LRAT). The results revealed significant acaricidal effects, with mortality rates of 77.7% and 82.2% for the Konyaalti and Kepez strains of the brown dog tick, respectively, in response to a 1% concentration of tar. LC_50_ and LC_90_ values were determined as 0.47% and 1.52% for the Kepez strain and 0.58% and 1.63% for the Konyaalti strain, respectively. When comparing the repellent effect of tar to the widely used synthetic repellent DEET, repellency rates of up to 100% were observed. As a result, this study establishes, for the first time, the larvicidal and repellent effects of *C. libani* tar on ticks.

## 1. Introduction

During our fieldwork in Antalya province of Türkiye (Turkey), where we investigated the acaricidal and repellent effects of plant-based products on ticks, we observed that local people sprayed an undiluted or diluted liquid called ‘katran’ extracted from the wood of a cedar tree (*Cedrus libani* A. Rich.) on domestic animals such as goats, sheep, and dogs. When we inquired about the reason for this, they mentioned that they have been protecting their animals against pests such as flies, fleas, lice, and ticks, especially by using katran (hereafter referred to as ‘tar’). According to the information we obtained from the interviews with local people, tar is added to the drinking water of animals several times a year by shepherds and local people. In addition, since ticks tend to attach to and live in certain areas of goats and dogs, such as the peri-anal region, abdomen, groin, feet, legs, and ears, these areas are treated at least twice a year, mostly in the spring and summer. This treatment is known to provide protection against ticks for a few weeks. In light of these observations and findings, we decided to test this dilute tar by obtaining it naturally to determine whether it has a killing and/or repellent effect on ticks (Acari: Ixodoidea), which are important ectoparasites of animals such as dogs and goats.

In many regions of the world, different types of tar are obtained from the old stumps of dead tree species belonging to the genera *Betula*, *Cedrus*, *Fagus*, *Picea*, *Pinus*, and *Juniperus*. The genus *Cedrus* in the Pinaceae family is represented by four species (*Cedrus atlantica*, *C*. *brevifolia*, *C*. *deodora*, and *C*. *libani*). The tar used in this study was obtained from *Cedrus libani* A. Rich, also known as Taurus cedar or Lebanese cedar. These names originate from the natural distribution of cedar in Türkiye, Syria, and Lebanon. The largest populations of *C. libani* in the world are in the southern regions of Türkiye. Currently, cedar forests in Lebanon are limited to small mountainous areas due to increasing human population activities [1].

The cedar tree and its products have been used by people for thousands of years in the construction of houses and ships, in the production of souvenirs, as food, and more. It was reported that oils and extracts from the wood, leaves, and seeds of the cedar tree are used as anti-cold, antispasmodic, and antimicrobial agents [2]. In addition, the tar from the Taurus cedar is used in internal and external treatments to accelerate wound repair, fight parasites, and alleviate various diseases in both humans and domesticated animals [3,4]. The tar used in this study was acquired through traditional techniques from the wood of cedar trees located in the Elmali district of Antalya. It is a fluid substance characterized by its unique pungent scent [5]. This liquid carries a notable smoky fragrance and presents an intense dark brown to black coloration. This naturally produced substance, which has biologically active properties, has been known to cure various problems for centuries. However, tar has a wide range of content, and the composition is affected by factors such as the specific extraction technique used, the age of the tree from which it is obtained, or the resin content in the wood [4,6,7].

Approximately 800 species of ticks have been identified worldwide, some of which serve as vectors for bacterial and viral diseases affecting human and animal health, such as Lyme disease and Crimean-Congo hemorrhagic fever [8,9,10]. Among the well-known tick species globally, the genera *Hyalomma*, *Ixodes*, and *Rhipicephalus* stand out. Ticks belonging to the *Rhipicephalus* genus are part of the hard tick family Ixodidae, with more than 70 species found worldwide. Studies conducted in various countries have reported that *Rhipicephalus* ticks are the most commonly found on vertebrate hosts, including humans, goats, dogs, and sheep [11]. Tick infestation can lead to a reduction in livestock body weight, milk production, and the development of anemia. Furthermore, tick bites can negatively affect the quality of animal skins. To combat this problem, various tick control strategies have been implemented. Among these methods, the application of antiparasitic acaricides to host animals such as cats, dogs, goats, and cattle is the most preferred approach. These acaricides can be applied to the skin through methods like dipping, spraying, or dripping, and they offer temporary protection against parasites, typically lasting from a few days to weeks [8,9,10,11].

Particularly, *Rhipicephalus sanguineus* sensu lato (s.l.) Latreille (Acari: Ixodidae) is known as the brown dog tick and acts as a vector for zoonotic disease pathogens such as *Rickettsia conorii*, *Ehrlichia canis*, and *Coxiella burnetii* [12]. The prevalence of these ticks in urban areas and parks raises concerns about human and animal health [13,14]. Unfortunately, recent studies have shown that *Rhipicephalus* ticks have developed resistance to chemical acaricides used by pet owners to protect against ticks in parks and gardens [15,16,17,18]. Additionally, the use of chemical acaricides poses significant risks to both animal and human health [19,20]. To address these concerns, scientists and local human communities around the world are actively researching and employing alternative products that are effective, do not harm human or animal health, and are less likely to induce resistance. In this study, we focus on the search for a new substance to protect humans and animals against ectoparasites. Therefore, it was aimed at determining the larvicidal and repellent effects of cedar tar obtained through traditional methods against the brown dog tick *R*. *sanguineus* s.l.

## 2. Results

The larval toxicity results of different concentrations of *C. libani* tar according to probit analysis on *Rh. sanguineus* s.l. collected from two different locations are presented in Table 1. When the LC_50_ and LC_90_ values obtained from two strains were compared, the Kepez strain was found to be more susceptible than the Konyaalti strain. The LC_50_ and LC_90_ values were determined as 0.47% and 1.52% for the Kepez strain and 0.58% and 1.63% for the Konyaalti strain, respectively (Table 1).

**Figure 1:**
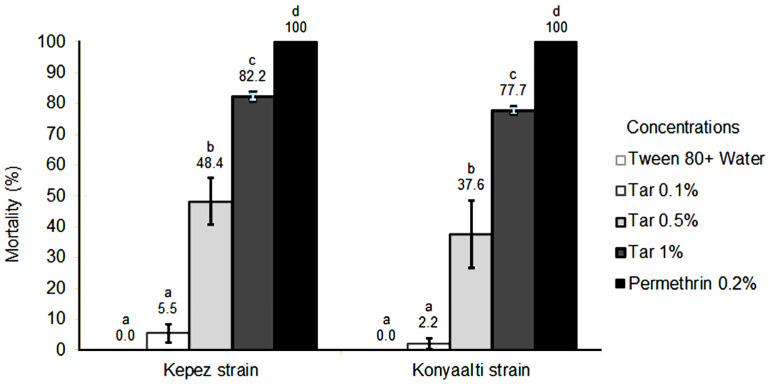
Larvicidal activity of *C. libani* tar compared on two strains of dog tick. Percent mortality averages were statistically compared for each strain using Duncan’s multiple range test (*p* ≤ 0.05). If the lower case letters are the same, there is no statistical difference.

When investigating the toxic effects of *C. libani* tar on tick larvae, it was observed that the tar induced varying levels of mortality. Figure 1 illustrates a graph displaying the mortality results and their statistical differences for different concentrations of *C. libani* tar on *Rh. sanguineus* s.l. collected from two distinct locations. From the graph, it was seen that the mortality rates ranged between 5.5% and 82.2% for the Kepez strain and between 2.2%, and 77.7% for the Konyaalti strain. Statistical analysis revealed that mortality rates were influenced by tar concentration. The concentrations used in both strains were not as effective as the positive control, Permethrin (0.2%), which exhibited a mortality rate of 100%. With the increase in concentration, a corresponding rise in the mortality rate was observed (Figure 1). The tar exhibited similar toxicity for both strains across all concentrations (*t*-test: *p* = 0.129).

**Figure 2:**
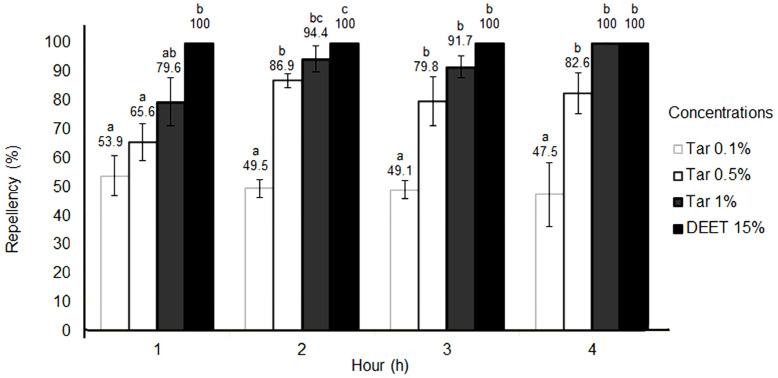
Repellent activity of *C. libani* tar compared with DEET on the Kepez strain of dog tick. Percent mortality averages were statistically compared for each strain using Duncan’s multiple range test (*p* ≤ 0.05). If the lower case letters are the same, there is no statistical difference.

**Figure 3:**
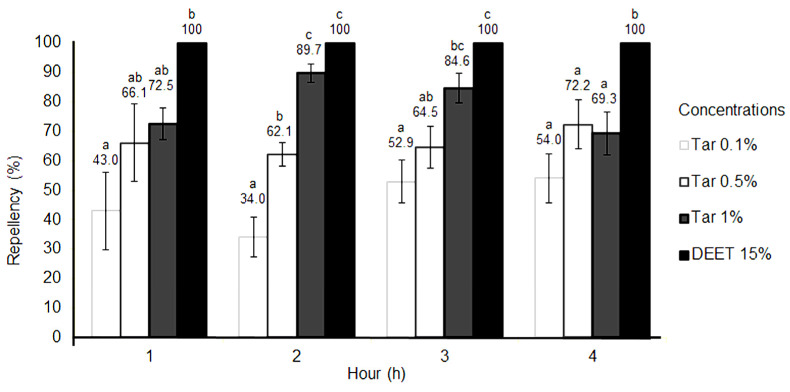
Repellent activity of *C. libani* tar compared with DEET on the Konyaalti strain of dog tick. Percent mortality averages were statistically compared for each strain using Duncan’s multiple range test (*p* ≤ 0.05). If the lower case letters are the same, there is no statistical difference.

The repellency results of the Kepez and Konyaalti strains at different observation intervals, along with their corresponding statistical differences, are depicted separately in Figure 2 and Figure 3. At the lowest tar concentration of 0.1%, the repellent effect on the Kepez strain ranged between 47.5% and 53.9%, while for the Konyaalti strain, it ranged from 34% to 54% (Figure 2 and Figure 3). At the end of 4 h, 0.5% concentration of tar showed a minimum 65.6% repellent effect on the Kepez strain. Meanwhile, the repellent effect rate for the Konyaalti strain at 0.5% tar concentration was between 62.1 and 72.2% at all times (Figure 3). At the end of the 4-h observation period, the Taurus cedar tar showed a high repellent effect varying between 79.6% and 100% on the Kepez strain at 1% concentration and was not statistically different (*p* > 0.05) at the 3rd and 4th h from the DEET, which was the positive control (Figure 2). For the Konyaalti strain, the repellent effect rate at the same concentration ranged between 69.3% and 89.7%. Statistically, the tar at 1% concentration was as effective as DEET at the 2nd h (Figure 3).

**Figure 4:**
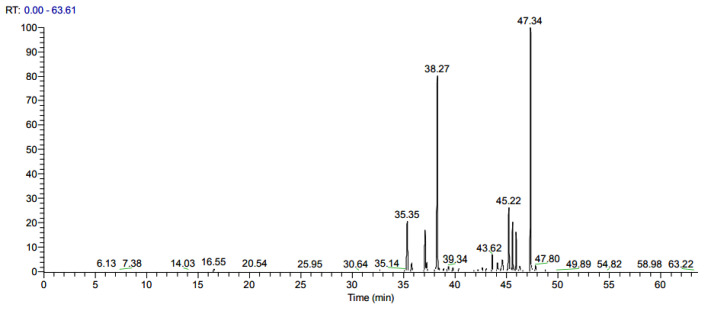
GC-MS chromatogram of *C. libani* tar.

The chemical composition of *C. libani* tar and its GC-MS chromatogram are presented in Table 2 and Figure 4. When the chemical content of Taurus cedar tar was analyzed, a total of 43 compounds were found, constituting 95.41% of the tar. The composition of the tar is primarily characterized by a diverse mixture of terpenes, with β-himachalene (29.16%), α-atlantone (28.7%), ar-turmerone (8.82%), longifolene-(V4) (6.66%), α-himachalene (5.28%), and β-turmerone (5.12%) emerging as the predominant constituents. Other constituents detected at more than 1% content were α-bisabolol (1.94%), veridiflorol (1.38%), glycl-l-proline (1.47%), and 1,2,3,4,4a,7-Hexahydro-1,6-dimethyl-4-(1-methylethyl)-naphthalene (1.19%).

## 3. Discussion

Concern about the negative effects on health caused by chemicals that people use in their daily lives, such as pesticides, as well as substances that come into contact with the skin like cleaning products, cosmetics, and insect repellents, is becoming increasingly common. On the other hand, findings on mutagenic, carcinogenic, and teratogenic effects resulting from the use of chemicals justify the concerns [21,22]. In order to reduce and prevent these adverse effects, scientific efforts to search for natural, rapidly biodegradable, and less risky products for health are increasing every day [23,24].

DEET, which we also used in our study, is one of the most widely used products worldwide to protect against mosquito-borne diseases such as malaria, West Nile Fever, Chikungunya, or Zika virus, and tick-borne diseases such as Lyme and Rocky Mountain spotted fever. Additionally, it serves to deter or repel many other species of ectoparasites from coming into contact with the body. DEET-containing products, available in various forms like sprays, lotions, liquids, and impregnated materials, have been marketed and used in nearly every region of the world since the 1950s [25,26]. While indeed effective, incorrect usage of DEET-containing products—such as disregarding label instructions—can lead to irritation of the eyes, lips, and sensitive areas, leading to redness and swelling. Accidental ingestion of these products can cause stomach discomfort, vomiting, and nausea [27,28,29]. Pets exposed to excessive amounts of DEET have exhibited reactions including vomiting, tremors, heightened excitement, and impaired coordination [30,31]. Due to the adverse effects associated with DEET, there is a growing trend in the utilization of plant secondary metabolites and plant-based products. An array of insect repellents is accessible, encompassing the well-known DEET as well as more recent ingredients like picaridin and the oil of lemon eucalyptus [32,33]. As can be seen, there is a need to search for alternative substances to ectoparasite repellent products that come into contact with human and animal skin. Our results show that cedar tar, with its repellent feature, is a candidate to meet this need. Cedar tar is also claimed by local residents to heal ulcer-like wounds when consumed diluted with water. In addition, various studies show that cedar tar has strong antibacterial [34] and antimutagenic [35] properties. With these properties, it can be said that cedar tar is also valuable to be examined in terms of wound healing and anti-infection effects.

In this study, *C. libani* tar was found to have repellent and lethal effects on dog tick larvae. It is quite gratifying that it exhibits a repellent effect comparable to DEET (15%) at a 1% concentration. The repellent activity test method (LRAT) employed in this paper is a simple, cost-effective, and safe testing approach. When compared to other methods used for assessing repellent effects, such as petri dishes and Y-tube olfactometers, the likelihood of ticks escaping from the test environment is nearly zero. This method allows for a large number of replicated experiments to be conducted simultaneously and requires very little space. Furthermore, there is no need to obtain permission from an ethics committee to conduct these tests [36]. Although the repellent properties of tar obtained from various trees using traditional methods or in the laboratory against ticks have been mentioned in our literature review, there are no findings indicating which tar, obtained from which tree, is effective against specific tick species and at what doses or concentrations. Therefore, our research constitutes the first larvicidal and repellency test of *C. libani* tar on ticks. However, essential oils obtained from the wood of some trees other than the *Cedrus* genus (e.g., *Juniperus*, *Calocedrus*) using diverse extraction methods like steam distillation or supercritical carbon dioxide extraction have demonstrated ovicidal, lethal, and repellent effects on ticks [37,38,39].

Some synthetic acaricides (e.g., Permethrin and Fipronil) applied topically to animals have protective effects against ticks. Permethrin, used as a positive control larvicide in this study, is used as both a repellent and a lethal agent against ectoparasite species such as sand flies and mosquitoes. When applied to uniforms, screens, and mosquito nets, it has killing and repellent properties for contact insects [40,41,42]. It is also used to manage *R. sanguineus* s.l. infestations in dogs [43]. In our study, 0.2% permethrin was found to be 100% effective against both strains of dog ticks. However, recent studies show that ticks have gained resistance to synthetic acaricides such as permethrin [15,43]. It is a satisfactory result that cedar tar is almost as lethal as permethrin against dog ticks at 1% concentration.

Furthermore, the larvicidal and repellent effects of essential oils obtained from various parts of different *Cedrus* species on certain insects, especially mosquitoes, termites, and some agricultural pests, have been investigated. The anti-termite activities of water:acetone (20:80) extracts of bark, sapwood, and both heartwood parts of Atlas cedar (*Cedrus atlantica* Manetti) wood were studied by Candelier et al. [44], who reported strong termite repellent activity. Furthermore, Atlas cedar oil was found to be toxic (lethal and deterrent) when administered to mealworm (*Tenebrio molitor* L.) larvae at different doses through the feeding method [45]. The essential oil of deodar cedar, *C. deodara* was tested for its insecticidal properties against the larvae of the diamondback moth, *Plutella xylostella* L. The oil exhibited varying levels of repellent, larvicidal, feeding deterrent, and growth inhibitory activities [46,47]. The larvicidal effect of essential oils obtained from the seeds of six populations of *C. libani* was investigated on *Culex pipiens* L., an important mosquito species for public health, and the oil exhibited high larvicidal activity, with LC_50_ values ranging between 47.8 and 116 ppm [48]. When *Cedrus deodara* essential oil-embedded pectin nanocapsules were applied to *Anopheles culicifacies* Giles larvae for four weeks, a remarkable mortality rate of 98% was achieved [49].

In this research, the repellent and lethal effects of tar on ticks could be attributed to the secondary components present in its structure and the synergistic interactions among these components. Studies have indicated that the composition of secondary metabolites in tar changes depending on factors like tissue type, extraction time, and environmental temperature [50]. Upon analysis of the results, it was determined that among sesquiterpenes, himachalol (22.5–32.4%), β-himachalene (21.17%), α-himachalene (5.9–10.5%), γ-himachalene (5.46–9.1), and α-atlantone (2.1–7.4%) are the primary constituents of cedar tar [5,51,52]. Singh and Agarwal [53] examined chromatographic fractions of Himalayan cedar wood oil (*C. deodara*) and studied the insecticidal impact of its major components, Himachalol and β-himachalene, on the housefly (*Musca domestica* L.) and the pulse beetle (*Callosobruchus analis* F.). The researchers observed a significant knock-down effect and lethal outcome in their experiments. Further, the essential oil obtained from *C. deodara* wood chips was found to be rich in himachalenes and atlantones, and it showed promising larvicidal activity against *P. xylostella* larvae [46]. Similarly, according to our analysis, himachalenes and atlantones account for more than 63% of the *C. libani* tar content. Our interviews with local residents report that tar is applied several times a year and is effective, but more research is needed to determine the duration of the tar’s lasting repellent effect on ticks. Although no adverse effects were observed when tar was applied to the skin of animals by herders or added to drinking water, further studies are needed on the off-target toxicity and bioaccumulation potential of tar in animals.

## 4. Materials and Methods

### 4.1. Extraction of Tar

Tar was obtained from the wood of the cedar tree (*C. libani*) using the traditional method of pyrolytic decomposition by local residents living in Elmali, a district of Antalya, Türkiye. Details of the extraction method can be found in Kurt et al. [5]. After extraction, it was stored in a refrigerator at +4 °C until its use.

### 4.2. Determination of Tar Composition

Volatile compound analysis in tar was conducted using a gas chromatograph with a mass spectrometry detector (GC-MSD). The analytical setup included a combination of advanced instruments, such as the Thermo Scientific TRACE 1300 gas chromatography, the Thermo Scientific ISQ 7000 mass spectrometry detector, and the Thermo Scientific RSH Triplus autosampler (Thermo Fisher Scientific Inc., Waltham, MA, USA). This setup was employed to detect the presence of terpenoid compounds within the tar. The chromatographic procedures were carried out using Xcalibur software (Version 4.2.47). An analytical column named TRACE TR-5MS (comprising 5% phenyl and 95% dimethylpolysiloxane, with dimensions of 30 m × 0.25 mm and a film thickness of 0.25 μm) was utilized for chromatographic separation, with all components originating from Thermo Fisher Scientific Inc.

To prepare the tar sample for analysis, it was diluted at a ratio of 1/500 with acetone and introduced into a 2 mL vial, which was subsequently injected into the GC-MSD instrument. The entire analysis process took 65 min. The inlet temperature was set at 250 °C, an injection volume of 2 μL was used, and a split ratio of 1/20 was applied. Helium gas was used as the carrier gas, flowing at a rate of 1.5 mL/min. The oven temperature was programmed to start at 30 °C for the initial 5 min, after which it increased at a rate of 5 °C/min until reaching 280 °C, where it was maintained for an additional 10 min. The detector temperature was set at 230 °C. For mass detection, the mass spectrometer operated in scan mode within the range of 40–550 Da.

To identify the compounds present, comparisons were made with spectra found in the Wiley 1n.l and NIST 0.5 databases from the National Institute of Standards and Technology. For each identified compound, a set of basic, molecular, and qualifying ions was selected as part of the identification process.

### 4.3. Identification and Storing of the Tested Ticks

Engorged female adult ticks belonging to two strains (Kepez and Konyaalti) of the *Rh. sanguineus* species complex were gathered without disrupting their rostrum from the ears and heads of domestic dogs in Antalya, Türkiye. The identification of ticks was carried out by the primary authors of this research using the taxonomic keys outlined by Aydin [54,55]. The collected ticks were maintained under controlled conditions with a temperature range of 26–28 °C, a relative humidity between 80–90%, and a photoperiod of 12 h of light followed by 12 h of darkness.

### 4.4. Larval Immersion Tests (LIT)

Following a two-week period, fully engorged female ticks laid their eggs. The emerging larvae, aged between 12 and 15 days, displayed upward climbing and host-seeking behavior in the holding tubes used for the experiments. The tar was dissolved in a solution of 0.3% Tween 80 (CAS No. 9005-65-6). Three distinct concentrations (0.1%, 0.5%, and 1%) of tar were used for the larval immersion tests. Over 50 larvae in the specified age range were gently placed on individual filter papers (7.6 × 8.9 cm) (Whatman No. 1), which were then folded and secured with clips to create packets. These packets were immersed in the designated concentrations for a duration of 5 min. Following the exposure period, the packets were taken out of the solutions and allowed to air-dry at room temperature. After 24 h, the packets were unsealed, and the survival status of the larvae was recorded. Larvae that did not respond to the contact of a paintbrush under a stereomicroscope were classified as deceased. All experiments were carried out in triplicate, and 0.3% Tween 80 solution was used as the negative control, 0.2% Permethrin (CAS no. 52645-53-1) solution was used as positive control group. The larvicidal activity tests were conducted at 24 ± 2 °C temperature, 50 ± 10% relative humidity with a photoperiod of 12:12 h light and dark conditions.

### 4.5. Larval Repellent Activity Test (LRAT)

**Figure 5:**
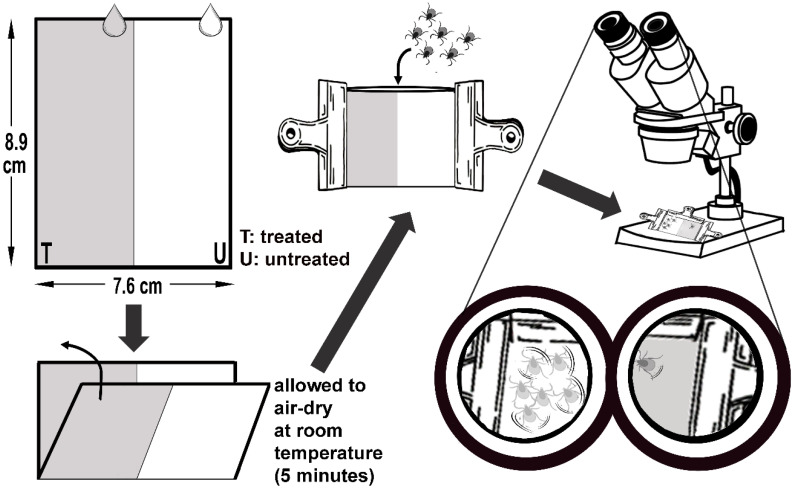
Illustration of larval repellent activity test (LRAT).

The repellent activity test method (LRAT) was developed by Koc et al. [36]. The same solutions and concentrations employed in the LIT assays were also used for the repellency test. A volume of 200 μL of the test solution was evenly and fully spread onto half of a Whatman filter paper (7.6 × 8.9 cm) using a pipettor, followed by a 5-min drying period before folding. Similar to the LIT procedure, more than 30 larvae within the age range of 12 to 15 days were placed on each treated filter paper. The filter paper was then sealed to form a packet (as illustrated in Figure 5). For comparison, Whatman filter papers treated with only 0.3% Tween 80 served as the negative control, while papers treated with a 15% (*v*/*v*) ethanolic solution of *N*,*N*-diethyl-*m*-toluamide (DEET) (CAS No. 134-62-3) acted as the positive control. The number of larvae staying in the treated or untreated sections within the packets was observed and recorded under a stereomicroscope at hourly intervals for a span of 4 h. Ticks were considered to be repelled if they remained in the untreated (control) part. All experiments were conducted three times. The percent repellency was calculated using the subsequent formula:

$$Percent\;repellency\;(\%)=\frac{\left[\left(Ticks\;in\;control\;area-Ticks\;in\;treated\;area\right)\right]}{\left[(Ticks\;in\;control\;area+Ticks\;in\;treated\;area)\right]}\times 100$$

The repellent activity tests were conducted at 24 ± 2 °C temperature, 50 ± 10% relative humidity conditions.

### 4.6. Statistical Analysis

All statistics of the collected data were assessed using SPSS 20.0 software. As per the Kolmogorov-Smirnov normality test, all data displayed a normal distribution (Sig. = 0.073; 0.200). Subsequently, the percentage means underwent analysis of variance (one-way ANOVA), and the means were contrasted using Duncan’s multiple range test (*p* ≤ 0.05). Lettering according to the statistical differences of the data is presented in the result graphs. The independent sample T-test was used to analyze whether two strain data (Kepez and Konyaalti) were different from each other. The larval mortality data were subject to probit analysis, and lethal concentration 50 (LC_50_) rates and lethal concentration 90 (LC_90_) rates with their confidence limits were determined.

## 5. Conclusions

It is clear that essential oils and tar extracted from tree species within the *Cedrus* genus exhibit various toxic effects on arthropod pest species. Notably, Taurus cedar tar demonstrates both repellent and lethal properties against the tick *Rh. sanguineus* s.l., a pest in both the public health and veterinary fields. Remarkably, Taurus cedar tar demonstrates comparable efficacy to DEET as a repellent and nearly equivalent performance to permethrin as a larvicide. This is the first study in which the repellent and larvicidal effects of *C. libani* tar on ticks have been recorded. This study emphasized the potential of cedar tar as an eco-friendly alternative for tick control, while further research is needed to understand its mechanism of action and effectiveness on other tick species.

## Figures and Tables

**Table 1:** Probit analysis of the larvicidal efficacy of tested *C. libani* tar against *Rh. sanguineus* s.l.

Strain	LC_50_ (%)	Confidence Limits (%)	LC_90_ (%)	Confidence Limits (%)	Chi-Square	*p*-Value
Kepez	0.47	0.40–0.55	1.52	1.20–2.12	1.38	0.239
Konyaalti	0.58	0.50–0.67	1.63	1.30–2.30	1.82	0.177

**Table 2:** Chemical composition of *C. libani* tar.

No	NIST Library Similarity (%)	Retention Time (min)	Substances	Composition Rate (%)
1	90.9	6.13	α-Pinene	0.09
2	70.7	7.66	m-Ethyl-toluene	0.09
3	81.2	10.47	1,2,3-Trimethylbenzene	0.06
4	72.3	12.31	m-Methylstyrene	0.06
5	75.9	12.88	p-Cymene	0.06
6	85.3	13.29	dI-Limonene	0.07
7	90.3	14.03	1-Methyl-4-isopropenylbenzene	0.21
8	90.5	15.46	Guaiacol	0.16
9	85.7	16.07	(2-Methyl-1-butenyl)-benzene	0.02
10	89.4	16.55	4-Acetyl-1-methylcyclohexene	0.53
11	84.7	18.84	3-Methylindene	0.03
12	81.3	19.79	p-Methylacetophenone	0.02
13	72.8	20.16	p-Menth-1-en-8-ol	0.03
14	81.6	20.54	Creosol	0.27
15	81.2	20.93	α,α,α-Trimethylstyrene	0.01
16	79.9	22.68	2-Methyl-1,2-dihydronaphthalene	0.02
17	91.4	25.95	p-Ethylguaiacol	0.10
18	81.9	26.92	α-Longipinene	0.04
19	74.8	28.31	2-Methoxy-4-propyl-phenol	0.03
20	91.1	30.64	Junipene	0.29
21	87.1	32.16	ç-Cadinene	0.16
22	92.6	32,82	Glcycl-L-proline	1.47
23	84.3	33.48	6-Methyl-2-p-tolyl-heptane	0.13
24	78.0	35.14	Exo-8-(2-Propeny)-endo-8-methyl-3-oxabicyclo[4.2.0]oct-5-ene	0.11
25	94.5	35.35	α-Himachalene	5.28
26	96.8	37.08	Longifolene-(V4)	6.66
27	74.2	37.24	1,2,3,4,4a,7-Hexahydro-1,6-dimethyl-4-(1-methylethyl)-naphthalene	1.19
28	87.6	37.61	α-Curcumene	0.12
29	84.4	38.06	ç-Muurolene	0.11
30	97.2	38.27	β-Himachalene	29.16
31	96.3	38.62	Cuparene	0.28
32	81.6	38.83	8,9-Dehydro-neoisolongifolene	0.46
33	86.4	39.34	9.10-Dehydro-cycloisolongifolene	0.54
34	88.0	39.78	α-Calacorene	0.10
35	82.6	42.71	cis-α-Bisabolene	0.38
36	85.6	41.93	Longiborneol	0.26
37	89.2	43.03	Dodecylbenzene	0.28
38	90.1	43.62	α-Bisabolol	1.94
39	80.9	44.10	Veridiflorol	1.38
40	82.8	44.49	α-Turmerone	0.57
41	81.4	45.22	ar-Turmerone	8.82
42	82.3	45.59	β-Turmerone	5.12
43	97.4	47.37	α-Atlantone	28.70
44			Others	4.59

## Data Availability

Data are contained within the article.
